# The changing roles of community nurses: the case of health plan nurses in Israel

**DOI:** 10.1186/s13584-017-0197-5

**Published:** 2017-12-23

**Authors:** Rachel Nissanholtz-Gannot, Bruce Rosen, Miriam Hirschfeld, the Community Nursing Study Group, Liat Arad, Shlomit Bloomenthal, Dorit Goldman, Yafa Haron, Miriam Hirschfeld, Calanit Kaye, Galit Kauffman, Idit Kogan, Rachel Nissanholtz-Ganot, Bruce Rosen, Bruria Sherman, Hedva Stoyer, Yulia Nigel, Dorit Weiss

**Affiliations:** 1 Department of Health System Management, Ariel University, University Hill, Ariel, Israel, 40700 and Myers-JDC-Brookdale Institute, JDC Hill, P.O.B. 3886, 91037 Jerusalem, Israel; 2 Myers-JDC-Brookdale Institute, JDC Hill, P.O.B. 3886, 91037 Jerusalem, Israel; 3 School of Nursing, Yezreel Valley College, Yezreel Valley, Israel

## Abstract

**Background:**

In Israel, approximately one-third of the country’s nurses work in community settings – primarily as salaried employees in Israel’s four non-profit health plans. Many health system leaders believe that the roles of health plan nurses have changed significantly in recent years due to a mix of universal developments (such as population aging and academization of the profession) and Israel-specific changes (such as the introduction of extensive quality monitoring in primary care).

**Objectives:**

The main objectives of the study were to identify recent changes in the roles of health plan nurses and their current areas of activity. It also explored the experience of front-line nurses with regard to autonomy, work satisfaction, and barriers to further role development.

**Methods:**

The study integrated interviews and surveys of nurses and other professionals conducted across 4 years. Data generated from earlier study components were used to guide questions and focus for later components.

In 2013, in-depth interviews were held with 55 senior nursing and medical professionals supplemented by interviews in mid-2017 with the head nurses in the four health plans. In addition, a national survey was conducted in 2014–5 among a representative sample of 1019 community nurses who work for the health plans and who are engaged in direct patient care. Six hundred ninety-two nurses responded to the survey, yielding a response rate of 69%. The survey sample consisted of an equal number of nurses from each health plan, and the observations were weighted accordingly.

**Findings:**

Senior professionals identified general themes associated with a shift in nursing roles, including a transition from reactive to initiated work, increased specialization, and a shifting of tasks from hospitals to community settings. They identified the current main areas of activity in the health plans as being: routine care, chronic care, health promotion, quality monitoring and improvement, specialized care (such as wound care), and home care.

In the survey of front-line nurses, 38% of the nurses identified “caring for chronically ill patients” as their main area of activity aside from routine care; 30% did so regarding “health promotion”, and 26% did so regarding “a specific area of specialization” e.g., diabetes, wound care or women’s health). In response to a separate question, 77% reported “great” or “very great” involvement in quality measurement programs.

Four out of five front-line nurses were satisfied with their work to a great or very great extent, and approximately three out of four of them (73%) felt that they had autonomy at work to a great or very great extent. About half of the nurses take into account, to a great or very great extent, the financial concerns of the health plans that employ them.

A large majority of the nurses (85%) indicated that the nature of their work had changed substantially in recent years, with an increase in autonomy noted as one of the key changes. Perceived barriers to further role development include attitudes on the part of some physicians and nurses, an insufficient number of dedicated nursing positions, and insufficiently attractive wage levels.

**Conclusions:**

The findings, gathered over 4 years, indicate alignment between universal and Israel-specific trends in health care and the evolving roles of nurses in Israel’s health plans.

The findings provide support for ongoing efforts in the health plans to give nurses more authority and responsibility in the management of chronically ill patients, a more central role in health promotion efforts, more advanced training - both inter-professional and nurse-specific, and more opportunity to focus on the roles and tasks that require nursing professionals.

## Background

The nursing profession is an important component of the provision of community healthcare world-wide. Nearly a third of the nurses in Israel are employed in the community,^1^ similar to the situation in the United States [2]. In Israel, most community-based nurses work for one of Israel’s four non-profit health plans, which are responsible for the organization and provision of care within the framework of Israel’s National Health Insurance Law [33]. Another major employer is the Ministry of Health (MOH), which employs nurses in its network of preventive mother and child health centers, and in other frameworks.

Traditionally, the main roles of community-based nurses (particularly in urban clinics) were to prepare patients for their meetings with a physician (by taking blood, pulse blood pressure, weight and other measurements) and implementing specific physician directives in the immediate wake of the patients’ encounters with the physicians. However, for over a decade, nursing and other leaders in Israeli health care have been talking about significant changes taking place in the roles of the community nurses. They have been attributing these role changes to a variety of contextual changes, which are described below. Interestingly, some of these contextual changes are happening in health care systems around the world, while others are Israel-specific. Some relate to changes in the population, other to changes in the health care system and still others to changes in the nursing profession itself.

### Changes in the population

With the **aging of the population**, there is a growing recognition among health care leaders of the need to better address chronic health problems [19, 22]. There is a considerable body of research demonstrating the substantial contribution that nurses can and do make to the management of chronic illness ([4, 5] CAN, 2013; Trehearne, Fishman and Lin, 2014). They improve quality and access to care [30] and play a central role in the provision of other types of health services that are particularly important to an aging population, such as home care and home hospitalizations.

### Changes in the health system

The increased attention being given by Israeli health plans to chronic care stems not only from population aging. Another motivator has been Israel’s **National Quality Monitoring Program** that has galvanized the plans to seek ways to improve the quality of care, particularly in the areas of chronic care and screening and prevention. Nurses are playing a central role in the health plans’ efforts to improve those measures [34].

Community nursing can play a major role in effecting life-style changes in chronically ill patients, in guiding direct caregivers in elder and childcare and in long-term care, and in identifying and preventing abuse [1, 9, 18, 20]. Moreover, various technological advances are making it increasingly feasible to manage the care of chronic patients and integrate the care across several geographic locations [3] and a range of different types of providers [7]; nurses play a central role in such integration efforts [8]. Interestingly, in some ways this a return to Israel’s 1980s “teamwork” model in which nurses played a significant role in chronic care.

Israeli health plans are also gradually recognizing the need to do more in the area of **health promotion** [14], well beyond those elements of health promotion related to the quality indicators. The growing health plan involvement in health promotion seems to stem from a mix of a mission-driven commitment to wellness, an interest in reducing health expenditures, and marketing considerations. Health promotion is clearly an area in which nurses are well situated to play important roles [13, 21]. Health promotion practices of nurses include disease prevention and health education; however, they seek to work in more collaborative way with other professionals [16].

The 2012 decision by the Israeli government to transfer the responsibility for **mental health services** from the government to the health plans has catalyzed health plan managers to also consider how various staffing models could help them address mental health needs in an efficient and effective manner ([32]: Nirel et al., 2007). In many countries, nurses play a major role in the provision of community-based mental health services ([11]; Heslop, Wynaden, Tohotoa and Heslop, 2016),), including identifying depression and other mental health problems and monitoring medication adherence/compliance. Note that the 2012 decision called for a 3-year transition period to prepare for a transfer of responsibility in mid-2015.

It is also important to note that **physician salaries** in Israel have increased significantly in recent years [33, 36]. There is a certain amount of overlap between the set of tasks that community-based physicians typically carry out, and the set of tasks that nurses are authorized to perform ([15]; Laurant, 2007). Accordingly, an increase in physician wages could encourage the health plans to shift certain responsibilities from physicians to nurses, and this in turn could contribute to both an increase in the number of nurses the plans employ and the scope of the nurses’ responsibilities.

### Changes in the nursing profession

Important changes are also taking place within the nursing profession itself. In particular, for several decades, the profession has been moving toward **greater academization and advanced specialty training** [15]. In recent years, various changes in primary legislation and Ministry of Health directives have broadened the scope of nursing practice, with increased authority in such areas as changing medication regimens, and palliative care.

At the same time, there are several **barriers to change** in the role of nurses in community settings in Israel. These include traditional perceptions of the role of nursing as “handmaiden to medicine” (and hence limited in autonomy), and opposition on the part of the Israel Medical Association to some of the changes being advocated by the nursing profession.

Interestingly, the major **nursing shortage** facing Israel [31, 23, 24] and countries around the world, could function either as a barrier to change (with the argument that basic functions be given top priority) or as a catalyst for change (due to the widespread belief that new and autonomous roles for nurses will attract more applicants to the profession). It will be important to see how these conflicting forces are playing out in Israel.

In several countries, major studies have been carried out to explore how such factors as the increasing importance of chronic, mental and preventive care are changing the roles of nurses in community settings [10, 17, 35]. Meanwhile, unique features of the Israeli health system, such as the pervasiveness of managed care plans, could result in a uniquely Israeli dynamic. However, until recently, no such studies had been carried out in Israel.

## Study goals

The study had two main goals:

- ♦ To document the main changes in the roles of health plan nurses in Israel in recent years
- ♦ To document the main areas of health plan nursing work today and the extent of their occupation in these areas.

- In addition, the study was designed to:
- ♦ Examine the extent of autonomy felt by nurses in their work
- ♦ Examine the extent of satisfaction felt by nurses with various aspects of their work
- ♦ Examine how nurses perceive their contribution to the quality measurement program and how the program affects them
- ♦ Identify the barriers to the continued development of the role of community nurses.

Apart from these issues, the study was designed to examine the varying perceptions of nurses based on the following variables: age, country of birth, level of education, professional status, and position of management or otherwise. Further, it compared the situation in Israel with that in the US and England regarding both how roles of community nurses have changed in recent years and the current nature of their roles.

The study focuses on nurses employed by Israel’s four non-profit health plans. Background information on the status of nurses in each of the four plans can be found in Appendix A.

## Methods

This paper is based on a broad study conducted in 2014 [28].

The study had two main components:

- In-depth interviews with approximately 50 leading professionals in the field of nursing and related fields.
- A survey of more than 1000 community nurses who engage in direct patient care

The two main components are complementary; the leading professionals are uniquely situated to identify the role changes that have taken place over time and to provide a broader system perspective, while the front-line nurses are uniquely situated to provide detail and quantification regarding the current nature and content of the work. Moreover, the in-depth, open-ended interviews with the leaders contributed to the development of the largely closed-ended questionnaire for the front-line nurses, (as did informal discussions with a number of front-line nurses). For example, the in-depth interviews generated a list of current areas of activity and then the survey was used to quantify the centrality of each area of activity and then explore it in greater detail.

These two main components are described further below; the study team also explored how the Israeli situation compares with the situations in the US and the UK, as described in Appendix B.

### A. Methods – The In-Depth Interviews of Leading Professionals

Fifty-five semi-structured, in-depth interviews were held with leading figures from the field of nursing and related fields. Interviewees were suggested by the members of the members of the Community Nursing Study Group (which included head nurses from the health plans and MOH nursing leaders). The interviewees included past and present heads of nursing at the MOH, the health plans, and the hospitals; leading academics in the field; managers at various levels of the health system (physicians, nurses and other personnel), the Israel Medical Association, the National Association of Nurses in Israel, hospitals, and various professional societies. The interviewees included both nurse managers and managers from other professions. The mix of interviewees made it possible to obtain a variety of perspectives on the processes affecting the nursing profession.

The interviews were conducted between January and August 2013. A semi-structured interview guide was used including questions on: the main current areas of activity of health plan nurses, how their work has changed in recent years, the challenges they face at work, their view of the future of the profession and of the barriers to changes to the profession.

The in-depth interviews were analyzed by first extracting the main themes and then exploring how these themes were addressed in the various interviews. Each interview was summarized in writing. An initial content analysis was carried out shortly after the interview, and significant issues were identified. In cases where a gap was discovered or a question arose, we returned to the interviewees and asked for clarifications.

In the analysis of the interviews, we compiled a detailed list of all the roles performed by the nurses and categorized them according to relevant groups. We made comparisons among health plans, different levels of management and regions. We focused on topics that were mentioned by many interviewees, but we also addressed noted significant issues raised by one or two interviewees. In addition, we analyzed reports and documents submitted to us by the interviewees.

In mid-2017, supplementary interviews were held with the head nurses in the four plans, focusing on changes in the most recent years, against the background of the findings from the survey.

### B. Methods – The Survey of Front-Line Health Plan Nurses

**The study population** consisted of all health-plan nurses in the four health plans spending most of their time in non-management work, regardless of whether their positions were full- or part-time. Registered nurses, Licensed practical nurses, and RNs with advanced certification were included. Nurses whose main occupation was managerial were not included in the survey to ensure that the focus was on nurses involved primarily in direct patient care. According to the data of the health plans, in 2014 this amounted to more than 4600 nurses.

#### Sample

Despite the differences in size between the four health plans in the number of employees and persons insured, approximately 250 nurses were sampled at random from each health plan to permit a reliable analysis for each plan.^2^

#### Data collection and distribution of the questionnaire

The research questionnaires were computerized and emailed to all the nurses using the addresses supplied by the health plans. The survey was conducted between August 2014 and February 2015. The appeal to the nurses invited them to respond by email through the link provided or by telephone.

In each health plan, 250 nurses were sampled randomly. This random sampling is relied upon to provide a representative mix of full v. part time, etc. within each health plan.^3^ At the data analysis stage, the observations were weighted to reflect inter-plan differences in sampling probability and response rate. Thus, the weighted sample can reasonably be expected to reflect the composition of the population of community nurses in Israel with at least some direct care responsibility – both in terms of health plan mix, managerial position mix, full-part time mix, etc.

The sample numbered 1019 nurses. Of these, 6.6% chose not to cooperate and 1.4% did not meet sampling criteria. In total, 692 or 69% of the nurses responded to the questionnaire.^4^ The response rate varied among the health plans, ranging from 56% to 82%. Four hundred forty-eight interviews were conducted by email; 175 – by telephone; and 69 were completed manually and posted by regular mail.

#### Research tool

The survey was composed of 70 questions, of which 15 were open-ended.

The topics covered included the following: the main areas of the nurses’ work; their main activities within those areas; perceptions of autonomy; perceptions of the quality measurement program; job satisfaction; perceptions of the professional future of health plan nurses; and background characteristics.^5^ A pre-test was conducted among 12 community nurses and the questionnaire was refined accordingly.

The categories for the question about usual work activities were derived from the leadership interviews.

The research questionnaires were computerized and emailed to all the nurses using the addresses supplied by the health plans. The survey was conducted between August 2014 and February 2015. The appeal to the nurses invited them to respond by email through the link provided or by telephone or regular mail. 65% of the responses were by e-mail, 25% by phone and 10% by regular mail. Comparison of key response variables (e.g. satisfaction, extent of role change, etc.) found very similar patterns of response across data collection modalities.

The questionnaire made it clear to the respondents that their responses would remain anonymous and the st.

udy team abided by that commitment, sharing with the health plans only aggregate results.

A key distinction in the survey was between routine work and “professional work”. Routine work was defined in the questionnaire as "work that is carried out routinely in the nurses' room, e.g., blood tests, ECG, blood pressure, vaccinations, inhalations, etc." Professional work was defined as "work in a specific professional field, such as treatment of complex wounds, stomas, follow-up in diabetes clinic, heart failure, giving instructions for genetic survey testing, geriatrics, women's health, mental health, day hospital, minor surgical procedures, etc.".^6^

#### Data analysis

The data were weighted to reflect differences among health plans in the sampling proportions and the response rate. After the weighting, the data presented unbiased national estimates of the parameters examined. The analysis focused on the findings for the full sample. Comparisons were also carried among population sub-groups defined in terms of age, educational level, and other background variables.^7^

The data were analyzed using SPSS version 21, and its complex samples utility. The open questions were analyzed using Naralyzer, a program that makes it possible to consider the entire set of responses to any given question. The responses were coded by category and a codebook was built for the questionnaire, after which the respondents’ responses were transferred to SPSS.

## Results

### A. Findings from the in-depth interviews of senior professionals

Senior professionals identified 4 key trends influencing the evolution of the work and functions of community and health plan nurses [26, 27]:

1. A transition from reactive to initiated work
2. Increased specialization of nurses in various areas of care, including diabetes and wound care
3. The integration of nursing work into various circles of care-giving and support (for example, with the patient as an individual, the patient within the family, and the patient within the group of patients suffering from the same condition).
4. A transfer of activities from the hospital to the community.

The general picture arising from the interviews is that the work of community nurses has broadened in recent years, and this trend is expected to continue.

The interviews identified the following as the main areas of current work of health plan nurses^8^:

- ♦ Routine work (as defined above)
- ♦ Care of chronic patients
- ♦ Health promotion (health education, encouraging screening tests etc.)
- ♦ Quality measurement and improvement (involvement in quality measurement programs in both her routine work and special projects).
- ♦ Home visits (Nurses perform most home visits, including assessments of pain and activities of daily living, functioning, care-giver teaching and support, treating wounds, changing catheters, and drawing samples for laboratory tests.)
- ♦ Specialized care in various areas including diabetes, stoma, treating wounds, and geriatrics
- ♦ Participation in unique health-plan initiatives such as “conversation maps for diabetes” and a “personal physician”.

The in-depth interviews also surfaced similarities and differences among the health plans; these are depicted in Appendix C.

Interviews in mid-2017 of the health plans’ nursing directors highlighted the following recent developments:

- With the support of top management, technical tasks are increasingly being delegated by nurses to support staff
- Nurses are increasingly being appointed to general management positions such as regional directors and program directors
- Inter-professional education, involving training of the full primary care team, is on the rise
- Nurses are increasingly being given patient management roles in chronic care
  and lead roles in health promotion and hospital-community transitions

### B. Findings from the Survey of Front-line Nurses

#### Overview

The main findings emerging from the survey are that the nurses feel that their role has broadened, that they enjoy substantial autonomy and, in general, they are satisfied with their work. They believe that the profession will continue to develop in the future, but at the same time, they point to significant problems and barriers.

#### Personal and professional characteristics

As indicated in Table 1, 93% of health plan nurses responding to the survey were women, 84% are Jewish, and 59% were born in Israel. Approximately 2/3 of respondents had academic degrees (47% bachelors and 17% masters), 90% were registered nurses (of whom almost half have taken advanced courses), almost half have been working as nurses for over 25 years (albeit not just in community settings), and about a third have supervisory or managerial roles.

**Table 1:** Distribution of respondents by key personal and professional characteristics (percents)

Personal characteristics		Professional characteristics	
Age		Educational level	
Up to 40	25%	Diploma (i..e. no degree)	36%
From 41 to 55	40%	Bachelors’ degree	47%
Over 55	35%	Masters’ or PhD degree	17%
Sex		Professional status	
Female	93%	Licensed practical nurse	11%
Male	7%	Registered nurse	50%
		RN with advanced course	39%
Population group			
Jewish	84%	Managerial responsibility	
Non-Jewish	16%	With managerial role	38%
		Without managerial role	62%
Country of birth			
Israel	59%	Nursing experience (years)	
Outside of Israel	41%	0–14	29%
		15–24	26%
		25+	45%

The study team compared the characteristics of the respondents and the study population for Israel’s two largest health plan, which account for more than half of the nurse population (similar data were unavailable from the other two plans). In one of the large plans, the characteristics were found to be very similar with regard to sex and education level, but with the sample having a slightly higher proportion of nurses who had done Diploma courses (54% v. 43%). In the other large plan, the population and sample were very similar with regard to sex, the proportion of the nurses who had done Diploma courses was slightly lower in the sample (33% v. 40%), and the sample also had a lower percentage of LPNs (8% v. 18%). (Note that the impact of these small differences on the overall study findings is apparently quite limited, as the study found few substantial differences in key study variables among these sub-groups – see Tables 5, 6 and 7 below).

#### Main areas of activity

A majority of front line staff (71%) continue to engage in routine activities (as defined in this study) to a great or very great extent. In addition, approximately one quarter to a third of the respondents indicated that their major area of care, besides routine work, was chronic illness care (38%), health promotion (30%), specialized care (26%) and home care (6%).

#### Caring for chronically ill patients

Those nurses who indicated that caring for the chronically ill was their main area of non-routine activity were presented with a list of relevant activities and asked to indicate in which of them they are involved to a great or very great extent. As indicated in Table 2, when it comes to chronic care, there is extensive involvement in several proactive and professionally demanding activities, such as management of the care process (80%) and interactions with specific patients (79%). About half also indicated great and very great involvement in the development of policy and general approaches to care regarding chronic patients. This process was also accompanied by the development of digital information systems and services.

**Table 2:** Percent of nurses who indicated that they were involved to a great or very great extent in various aspects of caring for the chronically ill

Management of the care process	80%
Pro-active interactions with specific patients	79%
Monitoring continuity of care for specific patients	79%
Development of policy and general approaches to care regarding chronic patients	48%
Outreach to the target population	52%
Development of proactive action plan for patients with chronic diseases	77%
Monitoring the implementation of proactive action plans	68%

(From among those nurses who indicated that caring for the chronically ill was their main area of non-routine activity)

The same group of nurses was also presented with a list of tasks associated with caring for individuals with chronic illness (Table 3) and asked whether the tasks constituted a significant part of their work. The vast majority affirmed that this was the case regarding healthy lifestyle counseling (91%) and deciding which patients to invite to visit (75%). In contrast, only 4% indicated that changing doses in prescriptions was a significant part of their work.

**Table 3:** Percent of nurses who indicated that various tasks were a significant part of their work

Healthy lifestyle counseling	91%
Functional and health status assessments	90%
Monitoring specific patients	86%
Deciding which patients to invite to visit	75%
Deciding, on the basis of protocols, computer alerts, and patient assessments whether/when particular patients should be immunized	63%
Changing doses in prescriptions	4%

(From among those nurses who indicated that caring for the chronically ill was their main area of non-routine activity)

#### Health promotion

Those nurses who indicated that health promotion was their main area of non-routine activity were similarly asked about the extent of their involvement in a list of relevant activities. As indicated in Table 4, there is extensive involvement in the identification of target populations for interventions (86%) and counseling about nutrition (79%), smoking (65%) and physical activity (73%). The nurses were much more likely to be involved in health promotion events within the clinic (38%) than in those outside the clinic (14%). When asked about the extent to which they engage in outreach (in general), 78% indicated that they do so to a great or very great extent.

**Table 4:** Percent of nurses who indicated that they were engaged in various activities related to health promotion, to a great or very great extent

Identification of target populations for interventions	86%
Workshops and counseling sessions in the area of health promotion	38%
Health promotion activities that take place outside the clinic	14%
Health promotion activities that take place in the clinic	44%
Counseling about nutrition	79%
Counseling about smoking	65%
Counseling about physical activity	73%

(From among those nurses who indicated that health promotion was their main area of non-routine activity)

#### Change in the way of working

Eighty-five percent of the nurses felt that concerning their main area of activity, in the past 5 years there have been significant changes in the way that they work. Among other things, the changes cited included: more planned work than in the past, focused activity on specific areas (such as the those noted above), and expansion of medical knowledge. Furthermore, 61% of the nurses noted that the working environment in this period had changed to a great or very great extent, and another 26% indicated that it had changed to a moderate extent.

#### Social and economic considerations

Almost all of the nurses (90%) take into account the family and economic situation of the patient to a great or very great extent while only 2% of them take it into account to a small or very small extent or not at all. Moreover, almost half (47%) of the nurses take into account financial considerations of the health plan to a great or very great extent while 18% of them take it into account to a small, very small extent or not at all. (The remainder takes these factors into account to a moderate extent.)

#### The quality measurement program

Approximately three-quarters of the nurses (77%) reported involvement to a great or very great extent in quality measurement programs; only 10% felt that they were not involved or were involved to a very small extent. Similarly, 75% of the nurses felt that the program had impacted their work to a great or very great extent, and 73% of them felt that their workload had increased to a great or very great extent as a result of the program.

#### Sense of autonomy

Approximately three-quarters of the nurses (73%) felt that they had autonomy at work to a great or very great extent. Similarly, 75% of the nurses felt that their professional autonomy had broadened in the preceding 5 years in contrast to 9% who reported a sense of curtailed autonomy in this period, and 16% who noted that they had felt no change. 70% indicated that, to a great or very great extent, they had the authority needed to do their jobs.

#### Work satisfaction

Four out of every five nurses (80%) were satisfied to a great or very great extent with their work overall, and only 3% felt little or very little satisfaction; the rest were moderately satisfied. In keeping with these feelings, 80% said that they would recommend to others to enter the profession.

As indicated in Chart 1, the aspects of work with which the most nurses were satisfied to a great or very great extent were: the impact of their work with patients (84%), the extent of responsibility given to them (81%), and their co-workers (82%). The aspects of work with which the lowest proportion of nurses were satisfied were: the salary (39%), physical conditions (14%, and appreciation of their work by their superiors (17% dissatisfied).

#### Barriers to the development of the nursing profession

Asked about barriers to the development of the profession, the front-line nurses who participated in the survey and the managerial nurses who participated in the in-depth interviews cited similar barriers. These included:

- ♦ **Physicians’ perceptions**. Some nurses believe that physicians fear the profession’s development, being interested in keeping the nurses in the position of following physicians’ directives.
- ♦ **Nurses**. Some nurses believe that they and/or some of their colleagues slow down the profession’s development, having no interest in broadening their authority for fear of a greater workload.
- ♦ **Lack of resources and positions**. Many nurses regard the lack of resources as a barrier to the profession’s development: the large workforce shortage, and the fact that there are no established positions
- ♦ **Compensation**. Subsequent to the relative wage dissatisfaction, some nurses regard low levels of compensation as a barrier to the profession’s development. Low compensation attracts fewer young people to the profession and increases the workload.

Note that the barriers identified in the survey were largely similar to those that came up in the in-depth interviews.

#### Differences among nurses related to background characteristics

The nurses’ perceptions were examined according to the following variables: age, country of birth, level of education, professional status and managerial position. As a rule, very few large or statistically significant differences were found among the groups (See Tables 5, 6 and 7). However, we did find that the more educated the nurses were, the more likely they were to take into account financial considerations of the health plan and the more satisfied they were than less-educated nurses. We also found that the higher the level of their professional training, the more they tended to take into consideration the family and economic situation of a patient. In addition, nurses with a higher level of professional training felt were more likely to indicate that the quality monitoring led to an increase in competition among the front-line clinicians, managerial pressure and overload.

**Table 5:** Authority and autonomy by sub-group

	Autonomy to a great or very great extent (in %)	Sufficient authority to carry out job (in %)
Total	78	70
Age		
Under 40	*70*	*60*
41–55	*80*	*72*
Over 55	*83*	*76*
Education level		
No degree	*84*	*84*
BA	*75*	*75*
MA+	*75*	*75*
Professional status		
LPN	*66*	*67*
RN	*78*	*72*
Beyond	*80*	*68*
Managerial responsibility		
With managerial role	*85*	*78*
Without managerial role	*74*	*65*

**Table 6:** Perceptions of significant change and level of satisfaction, by subgroup

	Significant change in past five years (in %)	Satisfied to a great or very great extent (in %)
Total	52	78
Age		
Under 40	*47*	*79*
41–55	*56*	*77*
Over 55	*52*	*80*
Education level		
No degree	*53*	78
BA	*54*	80
MA+	*43*	79
Professional status		
LPN	*61*	*71*
RN	*51*	*81*
Beyond	*49*	*77*
Managerial responsibility		
With managerial role	*62*	*81*
Without managerial role	*45*	*78*

**Table 7:** Social and economic care considerations by subgroup

	Significant weight given to family and economic circumstances	Significant weight given to health plan financial considerations
Total	90%	47%
Age		
Under 40	84%	38%
41–55	95%	55%
Over 55	84%	46%
Education level		
No degree	95%	44%
BA	86%	46%
MA+	88%	55%
Professional status		
LPN	81%	51%
RN	91%	46%
Beyond	89%	48%
Managerial responsibility		
With managerial role	89%	38%
Without managerial role	88%	55%

## Discussion and conclusions

This study provides the first empirically based description of the role of health plan nurses in Israel. Interviews with national leaders in nursing and medicine conducted in 2013 foreshadowed changes described by front-line nurses in interviews 2 years later. Leaders identified emergent trends in chronic illness and specialized care in the health plans as well a shift from reactive to nurse-initiated work. Findings from the 2014–5 survey of front-line nurses showed these trends were indeed occurring. Follow-up interviews with nurse plan leaders in 2017 provided key updates and were consistent with the earlier findings. All study components indicated that major changes have occurred in Israel in the role and functions of health plan nurses and their involvement in patient care.

The changes found included a transition from reactive to initiated work and increased specialization. Moreover, aside from routine care, the study has documented the central place of chronic care; health promotion and specialized care in the work of health plan nurses. Activities related to the quality monitoring and improvement are another significant component of the work of Israel’s health plan nurses. The findings provide support for ongoing efforts in the health plans to give nurses more authority and responsibility in the management of chronically ill patients, a more central role in health promotion efforts, more advanced training - both inter-professional and nurse-specific, and more opportunity to focus on the roles and tasks that require nursing professionals.

One of the study’s key findings is that about half of the nurses take into account, to a great or very great extent, the financial concerns of the health plans that employ them. This may be due, in part, to the growing roles of nurses in care coordination and management. This sensitivity to cost considerations, combined with the nurses’ broad professional expertise and patient-centric perspective, make nurses an extremely valuable resource for the plans, and positions them for greater responsibility and authority.

We examined whether there were differences between the nurses who are managers and the other nurses with regard to the extent to which they take into account various considerations. The two groups were equally likely to take into account the patients’ economic circumstances (approximately 90% do so to a great or very great extent). Surprisingly, however, the nurse-managers were less likely than the others to report that take into account the economic interests of their health plans (22% vs. 36%). The reasons for this difference might be a good subject of a future study.

Another key finding is that a significant proportion of the nurses (38%) indicated that care of the chronically ill patient is their main area of activity beyond routine care. With the population continuing to age, work in this area is likely to expand. It will be important to develop patient-centered projections of the amount and nature of the work that will be needed to care effectively for the chronically ill, and then think strategically about how health plan leaders, and physicians, nurses, and other professionals on the front-line can best work together to address this important challenge. This will clearly have important implications for the number of professionals required and continuing education, and this could be an important area for inter-professional continuing education. Serious consideration should be given to continuing the trend of giving nurses a greater role in care coordination and management of the chronically ill.

Health promotion is the main area of non-routine activity for another large group (30%) of health plan nurses. In the future, it will be important to explore in greater detail in which health promotion challenges they are most involved, the nature of their involvement in those challenges, and the degree of their success in those areas. This could have important implications for ensuring that nurses have the opportunity to engage in relevant continuing education opportunities regarding health promotion strategies, concepts and techniques. The major role that nurses are playing in patient-level health promotion should also be give greater recognition.

The study also confirms the extensive involvement of nurses in quality monitoring and improvement efforts, hence another area where inter-professional education should be given greater emphasis. A related finding is that almost all the nurses are quite attuned to the family and economic situation of the patient. This attention to context can be vitally important in efforts to improve a health plan’s quality performance, particularly with regard to those dimensions of quality, which require patient compliance and/or behavioral change. They will be even more important in the future if/when quality measures are developed that assess quality in such areas as depression care and community-based long-term care.

As indicated earlier, this study found that nurses perceive quality monitoring and improvement to be an important part of their work, but that this comes with various costs – more managerial pressure, more competition and a higher workload. A previous study by Nissanholtz et al. adds valuable context to these findings. In a national representative survey of health plan primary care physicians, most respondents (74%) agreed that nurses contribute to practice quality and share responsibility for improving quality measures. Physicians who felt that quality monitoring improved the quality of care and those who supported the program were more likely to consider that nurses shared responsibility for the quality of care [26, 27]. That study also found that, like nurses, the physicians also experienced the quality monitoring to increase managerial pressure, but to a lesser extent than did the nurses (58% v. 84%). Similarly, it found that physicians also felt that the quality monitoring increased competition, but to a lesser extent than did the nurses (47% v. 81%). The two professions were more similar in the extent to which they felt that the monitoring increased the workload (64% and 72%) [25].

The findings also indicated that a very high proportion of the health plan nurses are very satisfied with their work overall (80%). However, the study also highlighted pervasive concerns with the shortage of nursing positions, wage levels and the attitudes of some physicians and nurses that constrains the professional development of health plan nurses. Expanding nurses' role is one of the factors that improve satisfaction of nurses [12]. Dealing with these problems would clearly contribute to the satisfaction and commitment of those already working as health plan nurses. It may also help attract additional young people to the profession.

A related finding is that the vast majority of the health plan nurses are significantly involved in both routine work (in which they function largely as supports for primary care physicians) and more specialized work (in which they typically have more independent roles). In theory, a mix of this sort could be either a source of frustration (related to time constraints and/or the complexity of role identity) or a source of satisfaction (related to task diversity). Unfortunately, we do not have information on how this mix of roles is affecting nurse satisfaction. What we do know is that the vast majority of health plan nurses are satisfied with their work and that, at present, for most of them work entails a mix of routine tasks and specialized work.

In general, there is a good fit between the study’s findings about the evolving content of the work of health plan nurses and the contextual changes highlighted at the beginning of this article. One important exception is that, despite the ongoing reform of Israel’s mental health care system, the nurses did not indicate that mental health care is an important component of their workload. This may be because the in-depth interviews were carried out in 2013 and the survey of front-line nurses took place in 2014; while these were subsequent to the 2012 government decision to transfer responsibility for mental health care to the health plans, the transfer itself did not go into effect until 2015. Interestingly, in 2017 presentations to the Mental Health Reform Administration, the health plans spoke about significant strides in involving nurses in mental health care and in the future it will be important to study systematically the extent and nature of this evolution.

The changes in the roles of community nurses has apparently been a gradual evolution spread over many years. Interestingly, the main changes emphasized by nursing leaders in the 2013 in-depth interviews were largely similar to those emphasized in the 2017 follow-up interviews. At the same time, several new points were made in the 2017 interviews, suggesting that this evolution continues; these include the appointment of several nurses to regional director positions, greater expose of the nurses to patient clinical data, and more inter—professional educational.

Overall, the data presented in this study can contribute to Israeli efforts in the following areas:

- ♦ **Recruiting more people to the nursing profession, especially to community nursing**. The data can enable recruiters to provide a clearer picture of the current content of the work (including its diversity), how that content is changing in exciting directions, and how satisfied community nurses are with their work.
- ♦ **Intra-organizational thinking** within the health plans and other relevant organizations Key topics including the place of nursing resources in the budgeting of community-based services, the provision of appropriate levels of compensation, the definition of nursing tasks, the division of labor between the various staff members and mechanisms for increased cooperation.
- ♦ **Sharing information with the public** on the changed role of health plan nurses. This could contribute significantly to the empowerment of both nurses and their patients and to cooperation between these two groups.

### Limitations

The main study limitation is that the interviews were carried out at only one point in time. Thus, the findings about changes in the nurses’ roles are based, in part, on the nurses’ ability to recall the substance of their roles several years prior to the interviews and their assessments of how those roles had changed. This limited the extent to which the changes in the roles could be detailed and quantified.

Another limitation is that no formal assessment was made of the reliability and validity of the questionnaire used in the survey of front-line nurses. However, it was carefully derived from the in-depth interviews of the 55 nursing and other leaders and also reviewed for face validity.

Finally, as indicated in the methods section, there may be small-moderate under-representation of some sub-groups of nurses. However, the impact of these on the overall study findings is apparently quite limited, as the study found few substantial differences in key study variables among these sub-groups.

### Directions for further research

While the current study focused on Israel, it highlights developments, trends and issues, which may be relevant to other countries as well. For example, in the U.S. a 2017 report by the Macy Foundation called for using RNs as significant contributors to health promotion efforts in primary care settings [6]. That report identified a number of goals, including: giving more emphasis to primary care in nursing schools, developing primary care specializations, and encouraging teamwork.^9^

The study team plans to be in touch with researchers in other countries to identify similar studies, compare findings, and reflect together on similarities and differences. One of the issues of particular interest is whether the predominance of health plans in Israel has resulted in a unique mix of roles for its community nurses and whether Israel might be a harbinger for other countries in the process of adopting more organized systems of community care.

Directions for further research within Israel include:

- An exploration of how the nature of the interactions between health plan nurses and physicians are changing and how both professional groups feel about it
- Qualitative research to further understand how front-line nurses experience the mix of traditional/routine and newer/proactive roles
- More detailed analysis of the survey data regarding such topics as nurse involvement in the quality monitoring program, change barriers and facilitators, similarities and differences across age groups, etc.
- An analysis of how nurses answer the challenge of caring for the mentally ill and persons with mental health problems
- The evaluation of new nursing roles as nurse practitioners and nurse consultants and their impact upon patients’ and families’ quality of care and quality of life
- An analysis of the current roles of the nurses in preventive mother and child health centers, and how these have changed over time
- An exploration of of Israeli models of physician-nurse teamwork, which were recently praised by the OECD [29], and are continuing

## Appendix A: The status of nurses in Israeli health plans

Israel has four health plans (Clalit, Maccabi, Meuhedet and Leumit), and they have several important features in common. They are all non-profits and all were established at least 80 years ago. They all are nation-wide in scope and all have more than half a million members. They also have sophisticated IT systems, with all primary care clinicians working with electronic health records.

At the same time, there are several important differences among the plans. These include their size; the age, income and geographic mix of their members; and the extent to which their physicians are plan employees v. independent contractors. There are also important differences with regard to plan history, culture and operating style. Most significantly, for the purposes of this study, there are difference with regard to the extent, and manner, in which the plans work with nurses in community settings.

Clalit, with 4.4 million members, employs approximately 3000 nurses in community settings; most of them work on a full-time basis. The dominant model of primary care in Clalit is a community clinic with an interdisciplinary staff that has overall responsibility for the health and health care of the members registered in that clinic.^10^ The physicians and nurses jointly manage the care of each patient, including both responsive and proactive care, with a clear division of responsibility between the two professions. They also take a population approach and seek ways to improve the care of groups of patients (defined in such terms as age, illness and ethnicity).

Maccabi, with 2.1 million members, employs approximately 1100 nurses; this is up from 650 four years ago. Almost all of Maccabi’s nurses are RNs and two-thirds hold academic degrees. Most of them work either in municipal/district branches or in the clinics of independent physicians registered in the personal physician program (PPP); others work in such settings as specialty clinics or national nurse-led call centers that operate 24/7. In the PPP they function primarily as case managers for chronic illnesses and preventive/outreach services. In the branches, their jobs entail both proactive and reactive work. In recent years, the nurses have been able to delegate many of the more technical and routine tasks to nurse assistants or other support staff.

Meuhedet, with 1.2 million members, employs 880 nurses; many of them work on a part-time basis. The number of nurses in the plan has doubled over the last 5–6 years. Almost all the nurses work in Meuhedet’s municipal/district branches, each of which has a large number of specialist physicians and nurses. This has facilitated a rapid movement toward greater specialization of the nurses, particularly in those clinical areas where nurses have substantial authority and independence. These include diabetes, stoma, genetic counseling, and most recently palliative care. In each of these areas, the nurses play a central role in integrating the work of different types of professionals (physicians, dieticians, pharmacists, etc.) and in managing the care of the patient. The need for integration has been a major driver toward greater specialization.

Leumit, with 0.8 million members, has almost 500 nurses, with about 60% of them working full-time. Approximately 90% of them are RNs. The number of nurses working in the plan has been stable in recent years. The nurses work predominantly in the clinics that are operated by the health plan and secondarily in home care units, rather than in IP offices. Their work has expanded from the more technical tasks to include a large component of chronic care management and patient education. Recently, a new nursing management level – the district head nurse – was created, with a focus on implementing and inculcating various guidelines related to nurses’ newer responsibilities.

Nurses also play a central role in health plan call centers, in which have they have a great deal of autonomy to make decisions which often have significant clinical and economic implications.

## Appendix B: An initial examination of community nursing roles in the US and England, in comparison with Israel

### Methods

Ten telephone conversations were held with key figures in the US and England and the relevant professional literature was reviewed. This was followed by a comparison with the situation in Israel as it emerged from the in-depth interviews.

### Findings

- **Changes over time**. The role of community nurses has expanded in recent years. However, it is difficult to estimate the pace of change or point to countries with more rapid change. This topic would seem to deserve a separate study.
- **Different types of nurses**. One prominent difference between Israel and the other countries examined is that the latter have a category of Nurse Practitioners – NPs. In the US and England, nurses in this category enjoy broader authority. Though not many nurses in England are defined as NPs, their numbers are steadily growing. In the US, there are more than 222,000. In Israel, the Ministry of Health has begun to recognize nurse practitioners only in recent years and this, too, only in select areas (such as palliative care, geriatrics and diabetes care). Thus, the situation in Israel is very far from that in the US and England.
- **Managing the care of chronic patients**. The main difference between Israel and the other countries in managing the care of chronic patients concerns the nurses’ scope of authority. In Israel, the authorization of nurses to prescribe medication is very limited and hardly implemented and they do not make referrals. In the US and England, there are differences based on a nurse’s status and training. Some nurse work only according to a physician’s directives while others work more independently, make referrals and prescribe medications.
- **Home visits**. In Israel, as well as in the other countries, home visits for bedridden patients are performed mainly by nurses. However, in the US and England, the nurses working in homecare do it exclusively. In Israel, on the other hand, most of the nurses work in homecare in addition to their work at the clinic.
- **Measuring quality**. Both of the countries examined have quality monitoring programs. However, whereas in Israel the program has for many years encompassed the entire community and virtually all nurses are involved in or affected – in the US, until recently, it applies only to organizations of managed care (about a third of the population). Recently, the federal government has instituted a program of quality reporting on care for the elderly; it is related to payment that has led to reporting from almost all primary care practices. Still, while nurses are involved in quality measurement programs in both Israel and the US, the extent of their involvement apparently differs. Moreover, in England and the US there are financial incentives for participating in these programs whereas in Israel there are none.
- **Authority to prescribe medication**. In both of the countries examined, nurses have partial authority to prescribe medication and the differences between the countries are reflected in the substance of their authority. Another difference relates to the extent that this authority is actually practiced. In these countries, nurses defined as NPs are authorized to prescribe medication. In Israel, virtually no nurses prescribe medication without a physician’s authorization.

Caution should be exercised in interpreting these differences between countries and their causes. Factors such as the structure of the system, cultural differences, and the milieu of the health system should be taken into account.

## Appendix C: Similarities and differences among the health plans that emerged from the in-depth interviews

The similarities related to the following aspects: a shortage of nurses, overall and for all districts except for the north; considerable advances in the technologies used by nurses; more studies and training than in the past; creativity.

Alongside the great similarity found between the nurses’ jobs in all the health plans, there were also several differences, including:

- **The implementation of the provision for special authorization**: Although the Ministry of Health widened the authority of community nurses, not all the health plans have implemented the provisions.
- **Integration of nurses into the measurement program**. There are differences in the extent of involvement and responsibility of community nurses in the measurement program, directed by the Ministry of Health and examining the quality of prevention, diagnosis, and treatment provided by the health plans. (The program is implemented by the Israel National Institute for Health Policy Research and contains indicators for the following areas: immunization, early detection of breast and colon cancer, treatment of diabetes, asthma and heart problems.) In three of the four health plans, nurses are responsible for some of the indicators and manage them on the local, district and national levels. In the fourth, they deal with the technical aspects of measurement, such as administering vaccines or inviting patients to come in for a mammography or other tests.
- **Integrating nurses into various levels of health-plan management**. In three health plans, nurses serve in senior positions of general management (i.e., beyond nursing): they are integrated into the management of health-plan branches and districts, and of the health plan as a whole. In one health plan, they manage only the nursing sector and are not part of the general management of the health plan.
- **Developing and focusing on areas of specialization**. Each health plan invests in different areas of specialization. Differences in specialization also exist among the districts of a single health plan.

### Perceptions of barriers to the development of the profession

Despite the great potential for professional development of community nursing, the interviewees identified barriers such as: a lack of established positions; physicians’ perceptions of the profession and their fear of encroachment by nurses into their areas of responsibility; some nurses themselves do not wish to broaden their roles or are afraid of carrying responsibility even when they do receive it; nursing schools focus more on hospital-related studies, dealing less with the community and its diverse work possibilities.

## Footnotes

1 This figure of one-third is based on data from the Central Bureau of Statistics Labor Force Surveys for the years 2012–2014. It is up from one-quarter a decade earlier.

2 The data appearing in this report do not distinguish between the health plans. However, each health plan has received, for internal use, a series of tables comparing the findings concerning it and the others.

3 Inadvertently, in one of the health plans, nurses with less than 5years of experience were largely excluded in the sampling process. However, sensitivity analysis indicates that this had only a very limited impact on the study findings. This is because there were only small differences in key study variables between nurses with less than 5 years of experience and the nurses with five or more years of experience. In addition, the nurse with less than 5 years of experience constitute a relative small part of the study population (between 18% and 25% in the three plans for which we have data).

4 The non-response was due to an inability to contact 17% of the sample, 7% who refused to cooperate, 6% who initially indicated that they would respond but did not follow through, and 1% who were found to be irrelevant to the study.

5 The background variables included age, gender, ethnic background (Jewish, Arab, Druze, etc.), country of birth, level of education, professional status, management role, and years in the profession. They were included because they were felt to be potentially related to key study variables such as work satisfaction and perceived professional autonomy.

6 Education, giving guidance to patients, and referrals were not defined as routine work, nor as professional work. Evidently, they should be included in the professional work of nurses and not as routine work, but we cannot say with certainty that all the nurses understood that the educational part, as noted, belongs to professional work.

7 The analyses presented in this article do not distinguish among health plans, as the health plans’ agreement to participate in the study was conditioned on a commitment from the study team not to publish plan-specific findings. Each health plan has been presented with the findings for the results for their own nurses, compared with the national results.

8 These areas are consistent with the major changes in the health care system. For example, the fact that people live longer, but with more chronic diseases, requires increased attention to chronic patients, both to prevent deterioration of health status through the promotion of health and to respond to patients who are disabled. The fact that nurses learn more and have more extensive knowledge and capabilities than in the past affects the subject of “specialization” in various subjects and the nurses’ desire to develop in the field closest to their hearts and improve their abilities in this field

9 This approach is a natural continuation of the IOM report on the future of caregiving (IOM 2011). The IOM report noted that the nursing profession’s focus on patient management and coordination, patient education and public health intervention, is a good fit for a world in which health promotion and care management are increasingly being emphasized.
